# Revision of the Japanese Association for Acute Medicine (JAAM) disseminated intravascular coagulation (DIC) diagnostic criteria using antithrombin activity

**DOI:** 10.1186/s13054-016-1468-1

**Published:** 2016-09-14

**Authors:** Toshiaki Iba, Marcello Di Nisio, Jecko Thachil, Hideo Wada, Hidesaku Asakura, Koichi Sato, Naoya Kitamura, Daizoh Saitoh

**Affiliations:** 1 Department of Emergency and Disaster Medicine, Juntendo University Graduate School of Medicine, 2-1-1 Hongo Bunkyo-ku, Tokyo, 113-8421 Japan; 2 Department of Medical, Oral and Biotechnological Sciences, University G D’Annunzio of Chieti-Pescara, Chieti, Italy; 3 Department of Haematology, Manchester Royal Infirmary, Oxford Road, Manchester, UK; 4 Department of Molecular Laboratory Medicine, Mie University Graduate School of Medicine, 2-174, Tsu, Mie Japan; 5 Third Department of Internal Medicine, Kanazawa University, Graduate School of Medical Science, 13-1, Kanazawa, Japan; 6 Department of Surgery, Juntendo Shizuoka Hospital, Juntendo University Graduate School of Medicine, 1129, Izunokuni-shi, Shizuoka Japan; 7 Recomodulin Strategy Planning Department, Pharmaceuticals Sales Division, Asahi Kasei Pharma Corporation, 1-105, Kanda, Tokyo, Japan; 8 Division of Traumatology, Research Institute, National Defense Medical College, Tokorozawa, Saitama Japan

**Keywords:** Disseminated intravascular coagulation, Sepsis, Antithrombin activity, Systemic inflammatory response syndrome, Anticoagulant

## Abstract

**Background:**

With advances in the treatment of sepsis, the systemic inflammatory response syndrome (SIRS) has been losing its prognostic power. Since the SIRS category is no longer used for the diagnosis of sepsis, the disseminated intravascular coagulation (DIC) diagnostic criteria released by Japanese Association for Acute Medicine (JAAM) should be modified. Thus, the purpose of this study was to examine the appropriateness of replacing the SIRS score with antithrombin activity in JAAM-DIC diagnostic criteria.

**Methods:**

We analyzed data from 819 septic patients who had received recombinant thrombomodulin. The relationships between the 28-day mortality rate and baseline laboratory and clinical parameters were examined using univariate and multivariate analyses, and the impact of replacing the SIRS criteria with antithrombin activity was evaluated.

**Results:**

The SIRS score, prothrombin time ratio, and antithrombin activity were associated with the 28-day mortality rate (*P* values = 0.013, 0.018, and 0.003, respectively, by multivariate analysis). A modified version of the JAAM-DIC diagnostic criteria using an antithrombin activity <70 % was capable of diagnosing the identical number (*n* = 706) and a similar severity of patients (mortality, 34.6 % versus 34.8 %).

**Conclusion:**

Since anticoagulant therapy is expected to be more effective in patients with more severe coagulation disorders, the modified version of the JAAM-DIC diagnostic criteria might be useful for discriminating patients with sepsis who are good candidates for anticoagulant therapy.

## Background

Recent advances in therapeutic strategies have significantly improved the outcomes of sepsis patients. The need for anticoagulant therapy for sepsis-associated disseminated intravascular coagulation (DIC) has long been discussed. A number of studies demonstrated that the efficacy of anticoagulant treatments such as antithrombin and thrombomodulin is limited to septic patients with concomitant DIC especially in the presence of severe coagulation disorders [1–5].

Among the criteria used to diagnose DIC, Japanese Association for Acute Medicine criteria (JAAM-DIC) have consistently shown higher sensitivity and predictive accuracy for mortality compared to other DIC criteria [6, 7]. One of the reasons hypothesized to explain the better performance of the JAAM-DIC was the inclusion of the systemic inflammatory response syndrome (SIRS) score within the JAAM-DIC. The SIRS was introduced as one of the criteria to diagnose sepsis in 1992 and has been used as a marker of disease severity [8]. In recent years, however, the prognostic relevance of the SIRS score has been questioned [9], and SIRS criteria have been omitted from the latest definition of sepsis proposed in 2016 [10]. Another problem associated with including the SIRS score in the diagnostic criteria for DIC was that this item does not directly reflect the presence of a coagulation/fibrinolysis disorder; thus, its use in DIC diagnostic criteria might no longer be appropriate. Thus, we intend to replace the SIRS score with another item to maintain the usefulness of DIC criteria in making decisions regarding the application of anticoagulant therapy.

Levels of antithrombin decrease in sepsis due to excessive thrombin generation [11], increased vascular leakage, and impaired synthesis and degradation by proteases [12, 13]. A lower antithrombin activity has repeatedly been reported in severe sepsis [14, 15] with a significant association with poor survival [16–18]. The measurement of antithrombin activity has become routinely available in most of the laboratories in Japan, and the Japanese Association for Thrombosis and Hemostasis (JSTH) proposed new DIC diagnostic criteria which include antithrombin activity (https://www.jstage.jst.go.jp/article/jjsth/25/5/25_629/_pdf). Despite the role of antithrombin in the coagulation cascade and the prognostic value of antithrombin levels in septic patients, none of the DIC diagnostic scores has evaluated the inclusion of antithrombin activity among the diagnostic criteria. In the present study, we replaced the SIRS criteria with the antithrombin activity in the JAAM-DIC diagnostic criteria and examined the characteristics of this new, modified version of the JAAM-DIC.

## Methods

### Data collection

The data set was obtained from a post-marketing survey of recombinant human soluble thrombomodulin (TM-α; Asahi Kasei Parma Corporation, Tokyo, Japan) performed by the Asahi Kasei Pharma Corporation between May 2008 and March 2010 [19] and was provided by the JSTH.

A total of 2516 Japanese patients with infection-associated coagulation disorder were registered in this survey; however, since the measurement of antithrombin activity was not mandatory in the protocol, a complete data set was obtained in 819 cases, and all of these patients were analyzed in this study. Many were admitted because of severe infection, but patients with sepsis were also included. All patients were treated with TM-α; patients who received any other anticoagulants prior to recombinant thrombomodulin treatment were excluded. Other exclusion criteria were as follows: patients with SIRS score ≦1, missing data for complete analysis, unknown outcome, hypersensitivity to TM-α, and pregnancy. The use of anti-platelets was permitted. The survey was conducted in accordance with the Declaration of Helsinki and Good Vigilance Practice and Good Post-marketing Study Practice.

### Laboratory measurements

The platelet count, fibrinogen/fibrin degradation products (FDP), prothrombin time (PT), and antithrombin activity were measured in local laboratories. To measure the antithrombin activity, the plasma anti-Factor Xa activity or the anti-thrombin activity was assessed (chromogenic substrate method, reference intervals: 70–120 %).

### Statistical analysis

The relationship between the 28-day mortality and each component of the JAAM-DIC criteria (i.e., SIRS score, platelet count, FDP, and PT ratio) and the antithrombin activity at baseline (day of DIC diagnosis) were examined by univariate analysis in a logistic regression model. Variables associated with 28-day mortality at a *P* level of less than 0.05 were analyzed using a multivariate analysis (standard method of logistic regression analysis). The analysis was conducted using the outcome (survived, 0; died, 1) as the criterion variate and the SIRS score, platelet count, PT ratio, FDP, and antithrombin activity as explanatory variates. The differences in mortality according to various antithrombin activities were examined using the χ^2^ test.

The numerical values in the text and tables are the median and interquartile range (IQR), unless otherwise noted. The results of the logistic regression analysis were reported as the odds ratio (OR), *P* values, and 95 % confidence interval (CI). For all the reported results, *P* < 0.05 was considered to denote statistical significance. The above-mentioned analyses were performed using JMP software, version 9.0 (SAS Institute Co, Ltd, Cary, North Carolina).

## Results

### Patient demographics

Among the 819 patients, 546 patients survived (66.7 %) and 273 patients died (33.3 %). DIC was diagnosed based on a score of 4 or more according to the JAAM-DIC diagnostic criteria, and 706 cases (86.2 %) fulfilled the JAAM-DIC criteria. Although TM-α treatment was usually initiated after the diagnosis of DIC, this was not strictly regulated and was independently decided by each physician. As a result, 113 patients did not fulfill the criteria of JAAM-DIC at the start of treatment. Table 1 shows the baseline characteristics of the patients. The median age of the survivors was 69 (56 − 78) years, while that of the non-survivors was 72 (62 − 80) years (*P* = 0.007). A significant gender difference was seen between survivors and non-survivors. Sequential organ failure assessment (SOFA) score, the requirement of mechanical ventilation, and the incidence of bleeding were higher in the non-survivors (*P* = 0.000, 0.000, and 0.030, respectively). The median SIRS score was lower in survivors than in non-survivors (*P* = 0.037). Regarding the coagulation profile, the platelet count was lower (*P* = 0.026), the PT ratio was higher (*P* < 0.001), and the antithrombin activity was lower in non-survivors (*P* < 0.001). The FDP was not significantly different between the survivors and non-survivors.

**Table 1:** Baseline characteristics of the patients

	Survivors (*n* = 546)	Non-survivors (*n* = 273)	*P* value
Age, years	69 (56 − 78)	72 (62 − 80)	0.007
Gender, male/female	285/261	175/98	0.001
Body weight, kg	51.8 (44.6 − 61.2)	53.0 (45.0 − 62.0)	0.580
DIC score	5 (4 − 7)	6 (4 − 7)	0.183
SOFA score	9 (6 − 12)	12 (8 − 14)	0.000
Mechanical ventilation, *n* (%)	160 (29.3)	159 (58.3)	0.000
SIRS score	3 (2 − 4)	3 (2 − 4)	0.037
SIRS score ≧ 3, *n* (%)	343 (62.8)	195 (71.4)	0.000
Bleeding symptoms, *n* (%)	69 (12.6)	50 (18.3)	0.030
Coagulation parameters			
Platelet count, ×10^9^/L	6.7 (3.8 − 10.2)	5.9 (3.2 − 8.9)	0.026
FDP, μg/mL	28.0 (13.2 − 50.1)	24.9 (11.1 − 44.6)	0.095
PT ratio	1.31 (1.17 − 1.50)	1.42 (1.22 − 1.70)	0.000
AT activity, *n* (%)	56 (44 − 70)	51 (36 − 65)	0.000

Continuous variables are given as median (interquartile range)

*AT* antithrombin, *DIC* disseminated intravascular coagulation, *FDP* fibrinogen/fibrin degradation products, *PT* prothrombin time, *SIRS* systemic inflammatory response syndrome, *SOFA* sequential organ failure assessment

### Factors associated with survival

Among the categories in JAAM-DIC, the univariate analyses showed that the patients’ baseline SIRS score (*P* = 0.019) and PT ratio (*P* = 0.002) were associated with the outcome. Among the coagulation profiles, the baseline antithrombin activity showed the strongest association with the outcome (*P* = 0.000). In contrast, a significant association was not observed between the platelet count and the patients’ outcome (*P* = 0.073) or the FDP and the patients’ outcome (*P* = 0.586). The significant associations between the outcome and antithrombin activity (*P* = 0.003), PT ratio (*P* = 0.018), and SIRS score (*P* = 0.013) were also confirmed by multivariate analysis (Table 2).

**Table 2:** Relationship between 28-day mortality and JAAM-DIC criteria at baseline

Item	Univariate			Multivariate		
	OR	95 % CI	*P* value	OR	95 % CI	*P* value
SIRS score	1.18	1.03 − 1.37	0.019	1.21	1.03 − 1.37	0.013
Platelet count	0.98	0.95 − 1.00	0.073	0.98	0.95 − 1.01	0.147
FDP	1.00	1.00 − 1.00	0.586	1.00	1.00 − 1.00	0.314
PT ratio	1.34	1.11 − 1.68	0.002	1.34	1.04 − 1.79	0.018
AT activity	0.99	0.98 − 0.99	0.000	0.99	0.98 − 1.00	0.003

*AT* antithrombin, *CI* confidence interval, *FDP* fibrinogen/fibrin degradation products, *JAAM-DIC* Japanese Association for Acute Medicine-disseminated intravascular coagulation, *OR* odds ratio, *PT* prothrombin time, *SIRS* systemic inflammatory response syndrome

Table 3 shows the OR of various factors to the outcome calculated using a logistic regression analysis. The OR of a SIRS score ≧3 and an antithrombin activity <70 % were 1.48 (*P* = 0.014) and 1.52 (*P* = 0.021), respectively.

**Table 3:** Relationship between the 28-day mortality, JAAM-DIC criteria, and antithrombin activity

	Cut-off value	Odds ratio	95 % CI	*P* value
SIRS score	≧3	1.480	1.080 − 2.027	0.014
Platelet count (×10^9^/L)	<120	1.242	0.821 − 1.880	0.304
	<80	1.277	0.938 − 1.739	0.121
FDP (mg/L)	≧10	0.803	0.554 − 1.164	0.247
	≧25	0.791	0.591 − 1.058	0.113
PT ratio	≧1.2	1.658	1.170 − 2.348	0.004
AT activity (%)	<70	1.522	1.065 − 2.176	0.021

*AT* antithrombin, *CI* confidence interval, *FDP* fibrinogen/fibrin degradation products, *JAAM-DIC* Japanese Association for Acute Medicine-disseminated intravascular coagulation, *OR* odds ratio, *PT* prothrombin time, *SIRS* systemic inflammatory response syndrome

### Comparison of mortality according to various antithrombin activities

The mortality increased as the baseline antithrombin activity decreased, and the mortality was significantly different at all cut-off values of 70 %, 60 %, 50 %, and 40 %. The mortality of the patients with a baseline antithrombin activity ≧70 % was 26.5 %, while that of patients with an antithrombin activity <70 % was 35.5 % (*P* = 0.021) (Table 4).

**Table 4:** Comparison of mortality differences according to antithrombin (AT) activity

	Mortality	*P* value
AT activity <40 %	45.5 % (85/187)	<0.0001
AT activity ≧40 %	29.8 % (188/632)	
AT activity <50 %	39.3 % (133/339)	0.0026
AT activity ≧50 %	29.2 % (140/480)	
AT activity <60 %	36.6 % (180/492)	0.0155
AT activity ≧60 %	28.4 % (93/327)	
AT activity <70 %	35.5 % (221/623)	0.0205
AT activity ≧70 %	26.5 % (52/196)	

### Comparison of original and modified JAAM-DIC diagnostic criteria

**Fig. 1:**
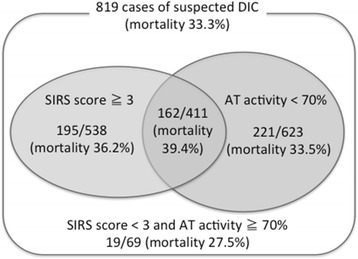
Comparison of patient distribution and mortality between subjects with a systemic inflammatory response syndrome (*SIRS*) score ≧ 3 and those with an antithrombin (*AT*) activity <70 %. Though the number of patients was larger in the category with an antithrombin activity <70 % than in the category with a SIRS score ≧3, the mortality rate was similar. The mortality rate in the category with an antithrombin activity <70 % was 35.5 %, while that in the category with an antithrombin activity ≧70 % was 26.5 %. The specificity for death was 72.2 % for the category with a SIRS score ≧3 and 73.5 % for the category with an antithrombin activity <70 %. *DIC* disseminated intravascular coagulation

The number of patients was 1.16-fold greater in the category with an antithrombin activity <70 % (*n* = 623), compared with the category with a SIRS score ≧3 (*n* = 538) (Fig. 1). However, the numbers of DIC patients identified by JAAM-DIC diagnostic criteria and the modified JAAM-DIC diagnostic criteria using antithrombin activity were exactly the same. The mortalities of these two criteria were almost identical (34.6 % versus 34.8 %) (Table 5).

**Table 5:** Number of patients diagnosed with DIC, SOFA score, and 28-day mortality in original and modified JAAM-DIC

		Number	SOFA score	Bleeding (%)	Mortality (%)
DIC	JAAM-DIC	706	7 (4 − 11)	14.7	34.6
	Modified JAAM-DIC	706	7 (4 − 11)	14.7	34.8
No DIC	JAAM-DIC	113	7 (4 − 11)	13.3	25.7
	Modified JAAM-DIC	113	7 (5 − 11)	13.3	23.9

*DIC* disseminated intravascular coagulation, *JAAM* Japanese Association for Acute Medicine, *SOFA* sequential organ failure assessment

## Discussion

There are three popular diagnostic criteria for DIC: the criteria of the Japanese Ministry of Health, Labour and Welfare (JMHW) [20], those of the International Society on Thrombosis and Haemostasis (ISTH) [21], and those of the JAAM [6]. The JAAM-DIC criteria were designed in 2006 to select candidates for anticoagulant therapy [22], and “applying the anticoagulation therapy from the early stage of DIC” was the fundamental concept [23]. Currently, antithrombin and recombinant thrombomodulin are the primary anticoagulants recommended in the Japanese guidelines [24]; however, some recent studies have demonstrated that the JAAM-DIC cannot discriminate candidates for recombinant thrombomodulin treatment [4, 25, 26]. Yamakawa et al. [25] reported that the probability of a beneficial effect from recombinant thrombomodulin increased as the baseline severity increased, and the JAAM-DIC category was not sufficiently stringent. Yoshimura et al. [4] suggested that recombinant thrombomodulin might be effective when the baseline APACHE II score was 24 or more.

Another problem with the JAAM-DIC criteria is the SIRS score. This item is no longer used for the diagnosis of sepsis [10]. Moreover, the SIRS score does not reflect coagulation/fibrinolysis disorders directly. Umemura et al. [1] demonstrated that anticoagulant therapies were effective in sepsis patients with coagulation dysfunction but not in those without dysfunction. In 2015, the JSTH proposed new DIC diagnostic criteria using the platelet count, PT ratio, FDP, some molecular markers, and antithrombin activity (https://www.jstage.jst.go.jp/article/jjsth/25/5/25_629/_pdf). In the JSTH criteria, the cut-off value for antithrombin was set at 70 % without any supportive data. Our data demonstrated that the mortality of patients with a baseline antithrombin activity of less than 70 % was 35.5 %, which might be sufficiently severe to warrant the use of recombinant thrombomodulin. Therefore, we adopted an antithrombin activity of more than 70 % as a cut-off value. As mentioned before, the efficacy of anticoagulants depends on the severity of the sepsis [4, 26, 27], and the efficacy of recombinant thrombomodulin was revealed in a population in which the post-treatment mortality was 20 % or more [4, 28], but it might not be efficient in a population with a mortality rate of less than 20 % [4, 26]. Thus, we think that the modified version of the JAAM-DIC might be suitable for identifying appropriate candidates for recombinant thrombomodulin treatment.

The present study had several limitations. First, many of the subjects analyzed in this study met the JAAM-DIC criteria at baseline. To propose new diagnostic criteria, an analysis of sepsis patients who might develop DIC is needed. Further validation should be done in subjects with or without DIC. Second, all patients included had sepsis-associated coagulopathy whereas other potential causes of DIC such as trauma, surgery, and burns were not evaluated. Future studies should examine the value of the modified scoring system in these DIC populations. Third, the cut-off value for antithrombin activity was empirically fixed at 70 %; however, the optimal cut-off should be determined using a larger number of subjects. Fourth, all the patients were treated with recombinant thrombomodulin. While it might be better to use data from subjects who did not receive anticoagulant therapy, such a study would be difficult to perform in Japan. Therefore, we utilized data from patients who uniformly received anticoagulant therapy as needed. Fifth, because fibrinogen and D-dimer data were not available, we could not compare the modified JAAM-DIC criteria with the ISTH or other diagnostic criteria. Such comparisons should be done in the future.

In summary, discriminating between simple coagulopathy and DIC is essential for deciding the proper timing of anticoagulant therapy. The modified JAAM-DIC diagnostic criteria were able to identify the patients who might benefit from anticoagulant therapy. The usefulness of these new diagnostic criteria should be examined in a prospective study.

## Conclusions

Since the SIRS category is no longer used for the diagnosis of sepsis, the JAAM-DIC diagnostic criteria should be modified. The replacement of a SIRS score ≧3 with an antithrombin activity level <70 % makes it possible to discriminate a more coagulation disorder-specific population. Thus, the modified version of the JAAM-DIC diagnostic criteria may be useful for identifying candidates for anticoagulant therapy among patients with sepsis.

## Key messages

- JAAM-DIC diagnostic criteria should be modified since the SIRS score has been losing its prognostic power and is no longer used for the diagnosis of sepsis.
- The replacement of a SIRS score ≧3 with an antithrombin activity level <70 % may make it possible to discriminate proper candidates for anticoagulant therapy among patients with sepsis.

## Abbreviations

CI: Confidence interval
DIC: Disseminated intravascular coagulation
FDP: Fibrinogen/fibrin degradation products
ISTH: International Society on Thrombosis and Haemostasis
JAAM: Japanese Association for Acute Medicine
JMHW: Japanese Ministry of Health, Labour and Welfare
JSTH: Japanese Association for Thrombosis and Hemostasis
OR: Odds ratio
PT: Prothrombin time
SIRS: Systemic inflammatory response syndrome
SOFA: Sequential organ failure assessment
